# In Silico Evaluation of Putative S100B Interacting Proteins in Healthy and IBD Gut Microbiota

**DOI:** 10.3390/cells9071697

**Published:** 2020-07-15

**Authors:** Massimiliano Orsini, Rosa Di Liddo, Federica Valeriani, Marzia Mancin, Renata D’Incà, Andrea Castagnetti, Antonio Aceti, Pier Paolo Parnigotto, Vincenzo Romano Spica, Fabrizio Michetti

**Affiliations:** 1 Istituto Zooprofilattico Sperimentale delle Venezie, Viale dell’Università, 10, 35020 Legnaro PD, Italy; morsini@izsvenezie.it (M.O.); mmancin@izsvenezie.it (M.M.); 2 Department of Pharmaceutical and Pharmacological Sciences, University of Padova, via Marzolo 5, 35131 Padova, Italy; rosa.diliddo@unipd.it; 3 Laboratory of Epidemiology and Biotechnologies, Department of Movement, Human and Health Sciences, University of Rome “Foro Italico”, Piazza Lauro De Bosis, 6, 00135 Rome, Italy; federica.valeriani@uniroma4.it; 4 Department of Surgery, Oncology and Gastroenterology, Gastroenterology Unit, University Hospital of Padua, 35121 Padua, Italy; dinca@unipd.it; 5 Wellmicro s.r.l, Via Piero Gobetti, 101, 40129 Bologna, Italy; andrea.castagnetti@wellmicro.com; 6 Clinical Infectious Diseases, Sant’Andrea Hospital, Sapienza University of Rome, 00189 Rome, Italy; antonio.aceti@uniroma1.it; 7 Foundation for Biology and Regenerative Medicine, Tissue Engineering and Signaling T.E.S. onlus Padua, Via De Sanctis 10, Caselle di Selvazzano Dentro, 35030 Padua, Italy; pierpaolo.parnigotto@unipd.it; 8 Department of Neuroscience, Università Cattolica del Sacro Cuore, Largo Francesco Vito 1, 00168 Rome, Italy; fabrizio.michetti@unicatt.it; 9 IRCCS San Raffaele Scientific Institute, Università Vita-Salute San Raffaele, 20132 Milan, Italy

**Keywords:** S100B, gut chronic inflammation, microbiome, bioinformatics

## Abstract

The crosstalk between human gut microbiota and intestinal wall is essential for the organ’s homeostasis and immune tolerance. The gut microbiota plays a role in healthy and pathological conditions mediated by inflammatory processes or by the gut-brain axes, both involving a possible role for S100B protein as a diffusible cytokine present not only in intestinal mucosa but also in faeces. In order to identify target proteins for a putative interaction between S100B and the microbiota proteome, we developed a bioinformatics workflow by integrating the interaction features of known domains with the proteomics data derived from metataxonomic studies of the gut microbiota from healthy and inflammatory bowel disease (IBD) subjects. On the basis of the microbiota composition, proteins putatively interacting with S100B domains were in fact found, both in healthy subjects and IBD patients, in a reduced number in the latter samples, also exhibiting differences in interacting domains occurrence between the two groups. In addition, differences between ulcerative colitis and Crohn disease samples were observed. These results offer the conceptual framework for where to investigate the role of S100B as a candidate signalling molecule in the microbiota/gut communication machinery, on the basis of interactions differently conditioned by healthy or pathological microbiota.

## 1. Introduction

S100B is a calcium-binding protein, which in the central nervous system is concentrated in astrocytes, although it is also expressed in other neural and extra-neural cell types [1]. Namely, the protein is also present, in addition to enteric glial cells, in retinal Muller cells, ependymal cells, oligodendrocytes, Schwann cells, some definite neuron subpopulations [2,3,4,5,6,7] and also in melanocytes, Langerhans cells, dendritic cells of lymphoid organs and some lymphocyte cell types, chondrocytes, Leydig cells, adrenal medulla satellite cells, skeletal muscle satellite cells, and adipocytes, which constitute an additional site of concentration for the protein [8,9,10,11,12,13,14]. When secreted, S100B is believed to have paracrine/autocrine trophic effects at physiological concentrations, but exhibits toxic effects at higher concentrations participating in inflammatory processes crucial for neural disorders, behaving as a damage/danger-associated molecular pattern molecule (DAMP) (for review, [1,15,16,17]). For enteric glia-derived S100B, neither acting mechanisms nor potential targets have been conclusively defined, although a role for the interaction of S100B with its receptor for advanced glycation endproducts (RAGE), has been shown in inflammatory processes of the gastroenteric tract [18,19]. Thus, the enteric S100B is considered as a diffusible cytokine that gains access to the extracellular space participating in immune-inflammatory processes in the gut [18,19,20,21,22,23].

Recently, the protein has also been shown to be present in human faeces, both in healthy and IBD subjects [24]. In the light of the consideration that cellular and molecular interconnectivity at this critical interface between the body and the environment is essentially unknown, S100B present in the intestinal wall and in faeces, and known to actively participate in physiopathological processes of gut mucosa, is a good candidate in playing a key role in the microbiota/gut communication machinery. Thus, the possibility of interactions between gut microbes and S100B protein appears of interest.

With the purpose of exploring this possibility, a study enlightening structural aspects of these potential interactions offers a useful prerequisite for further investigations. Indeed, S100B acts as a dimer or multimer of higher order [25], and it consists of two well-defined protein domains whose interactions are well investigated, namely the EF-hand and the S100 domain (for review, [26]). The Pfam database [27] reports known interactions with more than 36 domains for the former and 15 known interactions for the latter. Consequently, the S100B protein is potentially able to interact with any protein containing one or more of the above-mentioned domains, then hampering or simply modulating its oligomerisation and/or interaction with natural partners (e.g., RAGE). The aim of this work is to investigate if these putative S100B partners are detectable in human gut microbiota, and if they are differentially distributed between healthy and IBD subjects.

## 2. Materials and Methods

**Figure 1:**
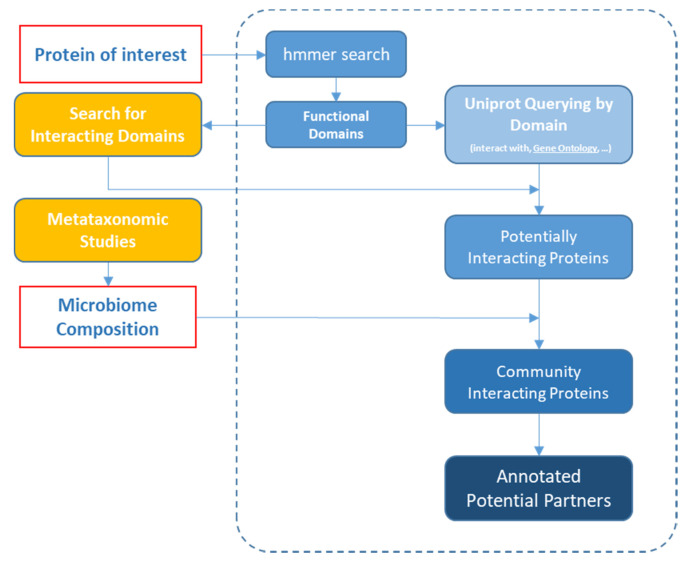
Implemented Analytical Workflow. A dedicated bioinformatic approach was developed to test in silico the possible interactions between a candidate protein and the microbiota proteome. The steps inside the dashed box represent the here implemented automatic workflow; data from the side (list of interactions and microbiome composition) have to be manually provided. A module for the automatic download of domain-domain interactions from the Pfam database is currently under development, but any alternative strategy can be applied to feed this automatic workflow.

A dedicated pipeline for identifying potential protein interaction partners of human S100B (Uniprot accession ID: P04271) has been implemented. The bioinformatics workflow was designed by integrating the interaction features of S100B known domains with the putative proteomics data derived from metataxonomic studies from gut microbiota (Figure 1).

The general approach was designed to realise a novel framework for evaluating interactions of a candidate protein versus the microbiota proteome S100B protein with the putative domains. Briefly, for a given protein, the functional domains are deduced by profile-sequence search using hmmer [28] and Pfam database [27]. The identified domains were searched through the iPfam database [29] to analyse all the potential interactions. The iPfam database considers the interactions between residues in three-dimensional protein structures (available in PDB [30]) and maps those interactions back to Pfam domains [27]. In parallel, the microbiome composition returned from metataxonomic studies of a population of interest, is used to filter out the Uniprot database retaining only those proteins belonging to the lowest taxonomic level available. A minimum annotation in terms of protein functional domains, cellular localisation and ontology terms are downloaded contextually. The obtained virtual proteomes are then filtered with the aim of retaining only those entries containing at least one target protein-interacting domain, as identified in the previous step. Pearson’s χ2 test was used to compare the frequencies of retrieved domains among groups. Optionally, resulting proteins could be further narrowed by cellular localisation or ontology features. For each protein, the annotation in terms of GO terms [http://geneontology.org] for the three categories was downloaded from the Uniprot database, and successively, the enrichment analysis was carried out using the Fisher’s test, implemented in the topGO R-package [31] and Rgraphviz [32] R-packages. Only those GO terms having an adjusted *p*-value (*q*-value) ≤0.01 were considered to be significantly enriched.

We applied it to the microbiota from patients affected by Ulcerative Colitis (UC, *n* = 8) or Crohn Disease (CD, *n* = 6) for which faecal S100B was measured using Human S100B (Protein S100-B) ELISA Kit, as previously described [24]. Briefly, faecal samples (25 mg) were dissolved in 2 mL of extraction saline buffer for 15 min at room temperature, and, subsequently, centrifuged at 12,000 *g* for 10 min. The patients’ samples were collected under the approval of the Ethics Committee of the University Hospital of Padua (protocol no. 46093/AO/2016). Bacteria were recovered by treating the biopsy with 2 cycles of ultrasound bath/vortexing (2 min) to disrupt the biopsy surface. After centrifugation (700 *g*, 1 min, 4 °C) to pellet debris, bacteria were collected from the supernatant by additional centrifugation (9000 *g*, 5 min, 4 °C); finally, DNA was isolated from the pellet using Qiagen DNeasy Blood&Tissue and quantified using the NanoDrop ND-100 spectrophotometer (NanoDrop Technologies, Wilmington, DE, USA). The hypervariable region V3-V4 of bacterial 16S rRNA was amplified using eubacterial primers [33], and sequenced with a MiSeq Illumina platform using manufacturer instructions for libraries preparation for 16S metataxonomic studies. The sequencing reaction was carried out on an Illumina MiSeq platform, and 250 bp paired-end reads were generated (Table S2). Control sequences were downloaded from the Short Reads Archive (SRA) [34] from a homogeneous sample of healthy adults of the same ethnicity and from the same sequencing platform. Finally, microbiome composition and diversity indexes were investigated by a full pipeline integrated into the QIIME2 package [35]. A detailed sample description and metagenomics analysis workflow has been provided in the Supplementary Material.

## 3. Results

The protein interaction pipeline (Figure 1) was realised and applied to investigate in silico the differences in the framework of putative partners of human S100B protein in the virtual gut microbiota of healthy and diseased people.

The hmmer analysis confirmed that S100B protein is composed of two functional domains: the S100 domain (AA 4–46) and the EF-hand domain spanning the residues 53–81. By the iPfam database, the former is potentially responsible of 9 domain-domain interactions, the latter could have interactions with 27 different functional domains, some of them in common with the S100 domain, then totally accounting for 32 unique protein domains (Table 1).

**Figure 2:**
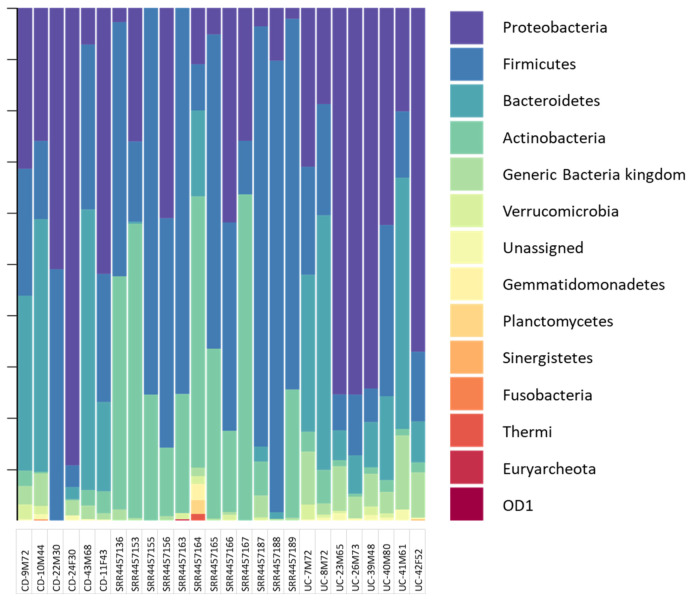
Microbiome composition. Bacteria community compositions at Phylum level, Relative abundance at phylum and genus level are in Supplementary Tables S3 and S4, respectively.

Bacterial communities from the gut of patients with active CD or UC were analysed together with 12 sequence datasets (Tables S1 and S2) from healthy individuals. The microbiome composition of both diseased and control sequences are reported in Figure 2 where they are summarised at the phylum level. Full details about microbiome composition and diversity indexes are reported in the Supplementary Material.

The overall differences between IBD patients and healthy subjects can be clearly appreciated. In particular, the dominant sequences in healthy subjects belong to Firmicutes and Actinobacteria phyla, accounting for over 97% of taxonomy. Among all dominant strains in IBD, Proteobacteria and Bacteroidetes were the most abundant (70–90%), followed by a significant fraction of sequences generically assigned to the Bacteria kingdom (Figure 2). Phylum level analysis also revealed subtle differences between CD and UC samples, where phyla as Fusobacteria and Planctomyces were observed in CD samples only, while OD1 and Synergistetes were detected in UC samples.

Metataxonomic analysis returned a total of 166 clusters of sequences for the three categories; for 74 it was only possible to assign unequivocally the taxonomy at genus level and the relative abundance in the all three groups of samples (Table S4), while other sequence clusters remained unassigned. Of the 74 genera, 15 were in common among the three groups of samples, 8 were found in UC patients only, 9 in CD patients, while 13 genus were specific of control samples (Table S4). The beta diversity analysis, showing the differences between microbial communities from different environments, highlighted that both UC and CD patients tend to cluster together with a high overlap between the two groups, suggesting a common bacterial community structure (Figure S1).

**Figure 3:**
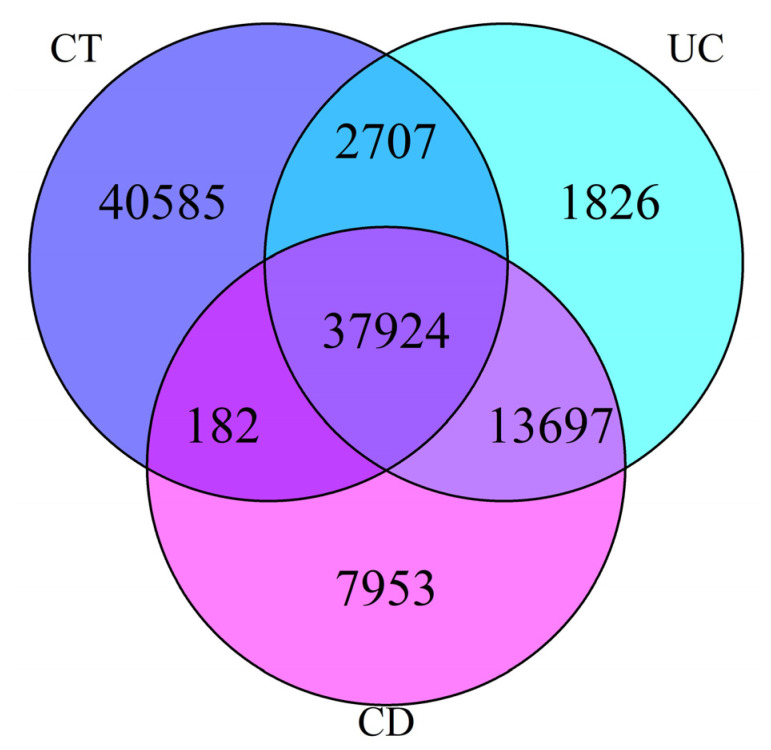
Distribution of S100B potentially interacting proteins in the three samples category. Abbreviations: CD—Crohn disease; UC—ulcerative colitis; CT—healthy control.

According to the proposed workflow, the Bacterial section of Uniprot database (*n* = 126,709,838 proteins) was queried using the above mentioned 74 genus (Table S4) describing the microbiome composition of both healthy and diseased people, providing a final pool of 32,344,661 candidate proteins (Table 2). These datasets were further filtered to retain proteins containing at least one domain potentially interacting with the human S100B protein then returning 61,086, 57,344 and 83,008 entries for the CD patients, UC patients and healthy controls (CT), respectively (Table 2, Figure 3). Interestingly, in the samples here used to validate the workflow, a decreased expression of S100B was observed in the faeces of CD patients (43.61–484.00 µg/L) and UC (27.37–937.54 µg/L) patients compared to healthy donors (65.22–2018.00 µg/L) [15].

Altogether these proteins consisted of 107,263 unique entries, differently occurring in the three categories (Table 2). The largest part (38.7%) of them appeared expressed by the healthy microbiome, a consistent fraction was theoretically expressed in all conditions (35.9%), and two smaller subsets appeared peculiar of the disease state, being specific in UC or CD diseased microbiomes, respectively (1.8%) and (7.6%) (Figure 3).

Going more in detail by exploring single functional domain distribution, it was possible to observe that some domains were not present in any of the microbiota proteome that were analysed (PF00340, PF02888, PF03520, PF08763, PF10163, PF12209, PF13833, PF00307, PF00531, PF00613, PF01023, PF05002, PF08457, PF11522, PF12424, PF12515); while other domains were displayed with a frequency close to zero (PF00167, PF00168, PF00992, PF01267, PF01372, PF01387, PF01576) and were not considered further, focusing only on the occurrence of the remaining domains as listed in Table 3.

Most of the selected putative domains showed an occurrence significantly lower than expected (*p*-value < 0.01), suggesting the presence of specific interactions. The Calcineurin-like phosphoesterase domain (PF00149) is the only domain overrepresented in all samples categories; conversely, the Helix-loop-helix typical of calcium-binding proteins (PF00036), the IQ calmodulin-binding domain (PF00612) and the EF-hand domain (PF12202) were found underrepresented than by chance. Interestingly, three domains were differentially represented in healthy versus diseased sample: namely, the Protein kinase domain (PF00069), the Immunoglobulin domain (PF13895) and the EF-hand_domain_pair (PF13499) were found overrepresented in healthy samples and underrepresented in all IBD sample groups.

In order to capture the function of the identified groups of proteins, the enrichment analysis was conducted for the three gene ontologies (biological process, BP; cellular component, CC; molecular function, MF). None of the three proteins groups showed enrichment for the BP category. Conversely, for molecular functions and cellular component categories, several differences among groups occurred. In the healthy group of proteins, the most enriched MF terms were: hydrolase, di- or tetra-phosphatases and phosphodiesterase, all of them showing a *p*-value <0.01. The same group in the CC category showed the integral component of the membrane as the most enriched but with a not significant *p*-value. These data could indicate hypothetical candidate pathways for further investigations, not in silico but experimentally validated.

Even for disease conditions, the bioinformatics approach provided candidate pathways. Proteins from CD diseased samples showed in addition, significant enriched MF terms related to nuclease and in particular exonuclease activities (Table 4).

Moreover, in the CC ontology, in addition to membrane related terms, several other specific terms related to cytoskeleton and movement appeared significantly enriched (*p*-value <0.05). Finally, the UC diseased samples showed an enrichment profile similar to the healthy samples in terms of MF category (hydrolase, di- or tetra-phosphatases and phosphodiesterase) without exhibiting any of the nucleases related terms observed in the CD samples. Similarly, in the CC category the UC samples showed enriched terms related to cytoskeleton and movement proteins (cytoskeleton, actin cytoskeleton, troponin complex, myofibril, contractile fibre) but differently from CD group did not show any terms related to the membrane protein localisation. On the contrary, CC ontology terms linked to the intracellular environment (organelle part, intracellular organelle part) were observed with a *p*-value <0.05. The observed data suggest a difference between CD and UC.

## 4. Discussion

The presence of S100B, both in the gut wall and in faeces suggests the possibility of an interaction between this protein and gut microbiome. This possible interaction is an obvious conceptual prerequisite for a function in this compartment involving together S100B and the gut microbiome. Due to its modular structure, the S100B protein is able to perform a variety of protein–protein interactions. In order to evaluate the hypothesis of possible molecular interactions between S100B and the human microbiome, we developed a bioinformatics approach (Figure 2) and applied it on the microbiome structure of healthy and UC or CD patients, to identify candidate target proteins for a putative interaction between S100B and microbiota proteome. The whole of the results supports the feasibility of a bioinformatics approach for studying in silico possible interactions between candidate proteins secreted in the gut and the microbiota proteome. We found potentially interacting domains both in healthy and IBD microbiome, conceptually supporting a potential interaction between S100B and gut microbes and, in addition, we also found apparent differences between the healthy and IBD groups. S100B is regarded to be associated with several diseases and neurological disorders where the microbiota also seems to play a role (for reviews, [17,23]). Further, in the faeces of patients with UC or CD, S100B levels were also shown to be altered [24] suggesting a dysregulation in these conditions also characterised by a microbiota dysregulation and an inflammatory process of the intestinal mucosa [36,37]. Moreover, the present study shows that gut microbiome protein domains potentially interacting with S100B are quantitatively reduced in IBD. Interestingly, in this respect, S100B in cultured enteroglial cells has been reported to be differently expressed after exposure to probiotic or pathogen bacteria [38]. This observation may support the hypothesis of a possible physiological role of S100B in healthy individuals, as well as a disruption of the putative interactive network in IBD condition.

To our knowledge, this is the first bioinformatics model developed to test a possible interaction between a secreted protein and the lumen microbiota proteome. The bioinformatics workflow was proposed to integrate a classical metataxonomic concept of community composition investigation with a domain-mediated protein-protein interaction approach.

The microflora composition of UC and CD patients was investigated together with a set of microbiomes from healthy people to obtain a taxonomic framework where to investigate proteins potentially interacting with S100B domains. Several differences were observed between the study groups and several candidate proteins were selected. Microbiota results are in agreement with other studies highlighting a strong community shift between healthy and disease state, and, as expected, the healthy microbiome was mainly composed by Firmicutes and Actinobacteria phyla while both UC and CD samples were dominated by Proteobacteria and Bacteroidetes [39,40,41]. These data strongly can impact on the composition of derived proteomes that, however, resulted comparable in size, further supporting the accuracy and suitability of the approach. Nevertheless, as above mentioned, this analysis does not have the purpose of a traditional metagenomics study on microbiota; but rather to obtain a rough microbial signature to narrow and compare the bacterial sets of proteins in a virtual in silico scenario. From the frequency distribution of functional domains emerged that in the virtual-gut proteomes the occurrence of S100B potentially interacting domains is feasible and, more interestingly, in the diseased samples the occurrence of such domains is significantly lower than in the control healthy group. It can be speculated that this may suggest a possible physiological role for S100B in the interaction between microbiota and gut mucosa, thus proposing S100B as a candidate signalling molecule. Thus, in IBD the S100B/microbiota interaction might even participate in pathogenic processes, such as chronic inflammation, which may involve TLR5 and/or TLR9, two recognised regulators of the mucosal immune response to microbiota [42]. Further, going down to a single domain level, it emerged that, differently from the general trend, some domains as PF00069 (Protein-kinase), the PF13499 (EF-hand domain pair) and the PF13895 (Immunoglobulin domain) are considerable over-represented in microbiome samples from healthy subjects respect than expected by chance, but above all, over-represented than in diseased samples (Table 3). This finding is consistent with the unexpected reduced levels of S100B in faeces of IBD patients in comparison with healthy subjects [24]. Although additional data will be needed in order to explain the finding, it seems to hint at a physiological role of S100B in the gut lumen, which is reduced in pathological conditions.

These in silico results open the way to experiments required for their confirmation in the laboratory. In any case, they induce several considerations and further working hypothesis. First of all, it can be speculated that in healthy microbiome more proteins can interact with the S100B, acting as a regulator and contributing in the switching off inflammatory processes or in physiological maintenance of the healthy mucosa. Conversely, in the disease state, the microbiota shift results in a qualitative change and quantitative reduction of exogenous S100B partners carried by the microbiota, then further promoting the inflammatory pathways or potentially influencing the gut-brain axis. The Gene Ontology enrichment analysis further shed light on the differences in the putative S100B interacting proteins in healthy and patients’ subgroups. Excluding the common signature of proteins related to hydrolase activity and membrane localisation, the targets derived from the CD gut proteomes were characterised by an exonucleases activity and a strong evidence of linking to bacterial membrane and cytoskeleton. Conversely, the proteins in the UC virtual proteomes did not show the nuclease activity nor the membrane localisation, but instead displayed an enrichment of cytoskeleton and intracellular organelle related terms. Interestingly, the interaction of S100B with the *E. coli* troponin, involved in bacterial division, was already reported years ago by Ferguson and Shaw [43].

The hereby proposed approach is based on a qualitative analysis and cannot exclude expression differences or microenvironmental interferences. Moreover, S100B is known to be a calcium-binding protein, although exhibiting calcium-binding affinities relatively weak compared to other members of the same EF-hand superfamily, and its calcium-induced conformational change is believed to facilitate the interaction with target proteins (for review, [44]). A different protein composition driven by selective expression of specific genes or differential abundance should be further considered by a complementary strategy. In this direction, shotgun metagenomics and transcriptomics studies can be helpful in improving the bioinformatics analysis. Indeed, the whole shotgun experiments and metatranscriptomics or metaproteomics can provide further valuable insights in functional features encoded by a microbiome [45,46].

## 5. Conclusions

A bioinformatics approach is a powerful tool for investigating the interactions between microbiota structure and human proteins. This in silico study addressed large amounts of bacterial proteins from metataxonomic studies from healthy and diseased people, in order to evaluate possible interactions virtually occurring between S100B protein and the respective microbiota proteomes. In particular, the present results offer the conceptual prerequisite to propose S100B as a candidate signalling molecule in the bidirectional microbiota/gut machinery interaction in health and disease. In principle, the same workflow could be applied to functional characterisation of other proteomic data further supporting biological basis to conceive a plausible interaction between a candidate protein and bacterial exogenous domains, then speculating a possible role in modulating physiological or inflammatory processes, as here reported for S100B protein and gut microbiota.

## Figures and Tables

**Table 1:** S100B potentially interacting domains. The S100B protein is composed of two structural domains: the S100 and the EF-hand. The table reports the list of protein domains potentially interacting with both of them. Some domains (e.g., the FGF domain) are able to interact with both. The S100 domain is also able to interact with itself generating homo-multimers.

Domain	Partner	Pfam ID	Description
S100B	C2	PF00168	Structural domain involved in targeting proteins to membrane
	EF-hand_1	PF00036	Helix-loop-helix domain or motif found in a large family of Ca-binding proteins.
	F-actin_cap_A	PF01267	Domain binding in a Ca independent manner the fast-growing ends of actin filaments
	FGF	PF00167	Fibroblast growth factors, Family of cell signalling proteins
	Ig_2	PF13895	Immunoglobulin domain
	IL1	PF00340	Interleukins
	Myosin_tail_1	PF01576	Myosin domain
	S_100	PF01023	S100 type calcium-binding domain
	SGS	PF05002	Structural domain found in calcyclin binding proteins
EF-hand	ATP_Ca_trans_C	PF12424	Plasma membrane calcium transporter ATPase C terminal
	CA_chan_IQ	PF08763	Voltage-gated calcium channel IQ domain
	CaATP_NAI	PF12515	Ca2^+^-ATPase N terminal autoinhibitory domain
	CaMBD	PF02888	Calmodulin binding domain
	CH	PF00307	Calponin homology (CH) domain
	Death	PF00531	Death domain
	EF-hand_1	PF00036	Helix-loop-helix domain or motif found in a large family of Ca-binding proteins.
	EF-hand_7	PF13499	EF-hand domain pair
	EF-hand_5	PF13202	EF hand
	EF-hand_6	PF13405	EF-hand domain
	EF-hand_8	PF13833	EF-hand domain pair
	EnY2	PF10163	Transcription factor e(y)2
	F-actin_cap_A	PF01267	F-actin capping protein alpha subunit
	FGF	PF00167	Fibroblast growth factor
	IQ	PF00612	IQ calmodulin-binding motif
	KCNQ_channel	PF03520	KCNQ voltage-gated potassium channel
	Melittin	PF01372	Melittin
	Metallophos	PF00149	Calcineurin-like phosphoesterase
	Myosin_head	PF00063	Myosin head
	PI3KA	PF00613	Phosphoinositide 3-kinase family, accessory domain
	Pik1	PF11522	Yeast phosphatidylinositol-4-OH kinase Pik1
	Pkinase	PF00069	Protein kinase domain
	S_100	PF01023	S100 type calcium-binding domain
	SAC3	PF12209	Leucine permease transcriptional regulator helical domain
	Sfi1	PF08457	Sfi1 spindle body protein
	Synuclein	PF01387	Synuclein
	Troponin	PF00992	Troponin

**Table 2:** S100B potentially interacting bacterial proteins in the three categories and their genus of provenance.

Category	Total Proteins	From Genus	S100B Interacting
CD	22,066,981	51	61,086
UC	21,402,684	50	57,344
CT	24,306,740	32	83,008

Abbreviations: CD—Crohn disease; UC—ulcerative colitis; CT—healthy control.

**Table 3:** The occurrence of S100B interacting functional domains over samples categories. The expected value by chance is reported. In the top those statistically significant (chi-squared Test, *p* < 0.01). In the bottom part, the domains not significantly supported (*p* > 0.5). Exp.: Expected Value.

Domain	CT	Exp.	CD	Exp.	UC	Exp.	Description
PF00036	35	110	16	100	17	97	Helix-loop-helix structural domain or motif found in a large family of calcium-binding proteins.
PF00069	35255	33790	16544	30657	13444	29731	Protein kinase domain
PF13895	149	76	12	69	10	67	Immunoglobulin domain
PF00612	8	20	9	18	9	17	IQ calmodulin-binding motif
PF00149	44022	43137	41838	39136	41095	37955	Calcineurin-like phosphoesterase
PF13202	1962	3421	2420	3104	2524	3010	EF hand
PF13499	2192	1226	696	1113	724	1079	EF-hand domain pair
PF13405	60	55	56	50	58	49	EF-hand domain
PF00063	2	3	2	3	2	3	Myosin head (motor domain)

**Table 4:** Gene ontology enrichment for bacterial candidate proteins, only terms showing a significant (*p* < 0.01) enrichment have been reported. Category: MF (molecular function), CC (cellular component). Terms: number of proteins annotated with a given GO term. Observed: number of proteins observed with a given GO term. Expected: number of proteins expected with a given GO term. Fisher: *p*-value according to the exact-Fisher test.

Category		GO Term			CD			UC			CT	
	GOID	Description	Annotated	Observed	Expect.	Fisher	Observed	Observed	Fisher	Observed	Expect.	Fisher
**MF**	GO:0016787	hydrolase activity	24136	20345	16818	<1e-30	18513	15394	<1e-30	15954	13142	<1e-30
	GO:0003824	catalytic activity	28253	20731	19687	<1e-30	18808	18020	<1e-30	16191	15384	<1e-30
	GO:0016462	pyrophosphatase activity	2508	2363	1748	<1e-30	2290	1600	<1e-30	1762	1366	<1e-30
	GO:0008796	bis(5′-nucleosyl)-tetraphosphatase activity	2155	2062	1502	<1e-30	1990	1374	<1e-30	1478	1173	<1e-30
	GO:0008803	bis(5′-nucleosyl)-tetraphosphatase (symmetrical) activity	2099	2014	1463	<1e-30	1950	1339	<1e-30	1436	1143	<1e-30
	GO:0004551	nucleotide diphosphatase activity	2156	2062	1502	<1e-30	1990	1375	<1e-30	1478	1174	<1e-30
	GO:0016818	hydrolase activity, acting on acid anhydrides, in phosphorus containing anhydrides	2517	2363	1754	<1e-30	2290	1605	<1e-30	1762	1371	<1e-30
	GO:0016817	hydrolase activity, acting on acid anhydrides	2519	2363	1755	<1e-30	2290	1607	<1e-30	1762	1372	<1e-30
	GO:0004527	exonuclease activity	911	848	635	<1e-30				663	496	<1e-30
	GO:0004518	nuclease activity	1004	856	700	<1e-30						
	GO:0016791	phosphatase activity	869	645	606	0.0016						
	GO:0004081	bis(5′-nucleosyl)-tetraphosphatase (asymmetrical) activity	55	48	38	0.0020				42	30	0.00065
	GO:0004721	phosphoprotein phosphatase activity	512				355	327	0.0044	452	279	<1e-30
	GO:0004115	3′,5′-cyclic-AMP phosphodiesterase activity	12				12	8	0.0045			
**CC**	GO:0016021	integral component of membrane	5163	161	155	0.0065						
	GO:0031224	intrinsic component of membrane	5163	161	155	0.0065						
	GO:0044425	membrane part	5165	161	155	0.0069						

## Supplementary Materials

The following are available online at https://www.mdpi.com/2073-4409/9/7/1697/s1, Figure S1: Beta diversity of the dataset microbiomes, Table S1: Dataset, Table S2: Reads count, Table S3: Microbiome composition at the phylum level, Table S4: Genus occurrence in microbiome samples categories, Supplementary Methods.
