# Supplementary material for: In Silico Evaluation of Putative S100B Interacting Proteins in Healthy and IBD Gut Microbiota
Source: Cells. 2020 Jul 15;9(7):1697. doi: 10.3390/cells9071697 (PMC7407188; doi:10.3390/cells9071697)
Supplement: Supplementary file 1 [file cells-09-01697-s001.zip › cells-805132_Supplementary material/SuppMat-30giugno2020_FigS1.docx]

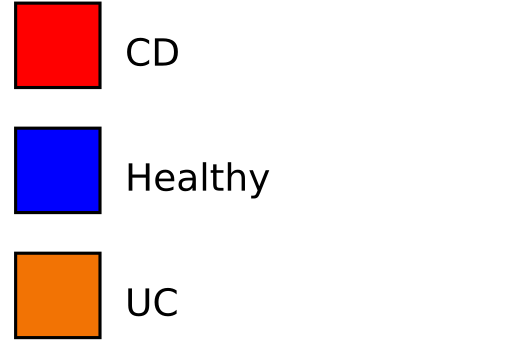

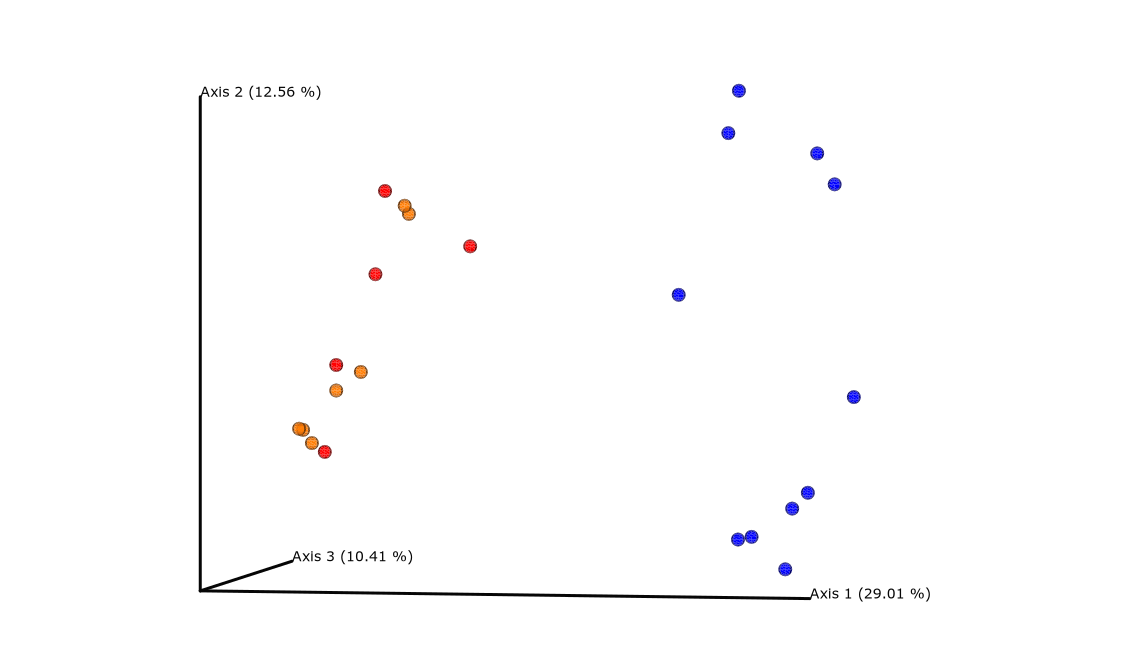


**Figure S1**. Beta Diversity. Patients and healthy subjects cluster in independent groups based on microbiota composition. Bray-Curtis metrics to compare differences in microbiota composition between CD, UC and controls. Bray-Curtis - takes abundance into account; non-phylogeny. Values are from 0 to 1. Principal Coordinate Analysis Plot (PCoA) is used for visualization of the data in the distance matrix in a 3D plot. The distance matrix is transformed into a new set of orthogonal axes where the first axis (Axis 1) can be used to explain the maximum amount of variation present in the dataset, followed by the second axis (Axis 2), third (Axis 3). Axes are different measures of variance calculated by QIIME2 software. The figure here reported is one of possible 3D orientations chosen to highlight differences among samples.
