# Supplementary material for: In Silico Evaluation of Putative S100B Interacting Proteins in Healthy and IBD Gut Microbiota
Source: Cells. 2020 Jul 15;9(7):1697. doi: 10.3390/cells9071697 (PMC7407188; doi:10.3390/cells9071697)
Supplement: Supplementary file 1 [file cells-09-01697-s001.zip › cells-805132_Supplementary material/SuppMat-30giugno2020_SMet.docx]

**Additional information on Methods**

**Dataset and metagenomics**

Fourteen patients, affected by Ulcerative Colitis (UC, n=8) of Crohn Disease (CD, n=6), were collected under the approval of the Ethics Committee of the University Hospital of Padua (protocol No. 46093). Bacteria were recovered treating biopsy with 2 cycles of ultrasound bath/vortexing (2 min) to disrupt biopsy surface. After centrifugation (700 g, 1 min, 4°C) to pellet debris, bacteria were collected from the supernatant by an additional centrifugation (9000 g, 5 min, 4°C); finally, DNA was isolated from pellet using Qiagen DNeasy Blood&Tissue and quantified using the NanoDrop ND-100 spectrophotometr (NanoDrop Technologies) The hypervariable region V3-V4 of bacterial 16S rRNA was amplified using eubacterial primers [1] and sequenced with a MiSeq Illumina platform using manufacturer instructions for libraries preparation for 16S metataxonomic studies. The library quality was assessed on the Qubit Fluorometer and the Agilent Bioanalyzer as indicated by the manufacturer. The sequencing reaction was carried out on an Illumina MiSeq platform and 250 bp paired-end reads were generated. A control group of sequences from 12 healthy adults obtained by human microbiota project [2] and downloaded from the SRA archive [3] were included to highlight differences in the community composition. In table S1 is reported the dataset.

**Data Analysis.** Microbiome composition was investigated by a full pipeline integrated in the QIIME2 package [4]. Briefly, paired end reads we demultiplexed and truncated by cutting off the barcode and primer sequence. Reads were trimmed by quality score and denoised by the deblur algorithm [5], multiple aligned by Mafft [6] and phylogeny deduced by FastTree [7]. In table S2 is reported the reads count. Sequences with > 97% similarity were assigned to the same operational taxonomic unit (OTU), then representative sequences were extracted. The representative sequences were taxonomically classified using the sklearn classifier [8] and the GreenGene database [9,10].

Then, per sample representative sequences and their abundance were used to calculate both alpha (observed_otus, Chao, ace, Shannon, Simpson, Faith); rarefaction curves were also evaluated. The microbiota data and community estimation were analyzed by unweighted unifrac analysis, Jaccard index and Bray-Curtis metrics to compare differences in microbiota composition between CD, UC and controls.

**S100B assay.** The presence of S100B in the feces of patients was measured using Human S100B (Protein S100-B ELISA Kit and stool, starting from 25 mg of feces dissolved in 2 mL of extraction saline buffer for 15 min at room temperature, and centrifuged at 10000 rpm for 10 min [11].

**Additional References.**

1. Klindworth A, Pruesse E, Schweer T, Peplies J, Quast C, Horn M, et al. Evaluation of general 16S ribosomal RNA gene PCR primers for classical and next-generation sequencing-based diversity studies. Nucleic Acids Res. 2013 Jan 7;41(1):e1.

2. NIH HMP Working Group, Peterson J, Garges S, Giovanni M, McInnes P, Wang L, et al. The NIH Human Microbiome Project. Genome Res. 2009 Dec;19(12):2317–23.

3. Kodama Y, Shumway M, Leinonen R, International Nucleotide Sequence Database Collaboration. The Sequence Read Archive: explosive growth of sequencing data. Nucleic Acids Res. 2012 Jan;40(Database issue):D54-56.

4. Bolyen E, Rideout JR, Dillon MR, Bokulich NA, Abnet CC, Al-Ghalith GA, et al. Reproducible, interactive, scalable and extensible microbiome data science using QIIME 2. Nat Biotechnol. 2019;37(8):852–7.

5. Amir A, McDonald D, Navas-Molina JA, Kopylova E, Morton JT, Zech Xu Z, et al. Deblur Rapidly Resolves Single-Nucleotide Community Sequence Patterns. mSystems. 2017 Apr;2(2).

6. Katoh K, Standley DM. MAFFT multiple sequence alignment software version 7: improvements in performance and usability. Mol Biol Evol. 2013 Apr;30(4):772–80.

7. Price MN, Dehal PS, Arkin AP. FastTree: computing large minimum evolution trees with profiles instead of a distance matrix. Mol Biol Evol. 2009 Jul;26(7):1641–50.

8. Pedregosa F, Varoquaux G, Gramfort A, Michel V, Thirion B, Grisel O, et al. Scikit-learn: Machine Learning in Python. Journal of Machine Learning Research. 2011;12(85):2825–30.

9. McDonald D, Price MN, Goodrich J, Nawrocki EP, DeSantis TZ, Probst A, et al. An improved Greengenes taxonomy with explicit ranks for ecological and evolutionary analyses of bacteria and archaea. ISME J. 2012 Mar;6(3):610–8.

10. Werner JJ, Koren O, Hugenholtz P, DeSantis TZ, Walters WA, Caporaso JG, et al. Impact of training sets on classification of high-throughput bacterial 16s rRNA gene surveys. ISME J. 2012 Jan;6(1):94–103.

11. Di Liddo R, Piccione M, Schrenk S, Dal Magro C, Cosma C, Padoan A, et al. S100B as a new fecal biomarker of inflammatory bowel diseases. Eur Rev Med Pharmacol Sci. 2020 Jan;24(1):323–32.
