# Supplementary material for: In Silico Evaluation of Putative S100B Interacting Proteins in Healthy and IBD Gut Microbiota
Source: Cells. 2020 Jul 15;9(7):1697. doi: 10.3390/cells9071697 (PMC7407188; doi:10.3390/cells9071697)
Supplement: Supplementary file 1 [file cells-09-01697-s001.zip › cells-805132_Supplementary material/SuppMat-30giugno2020_TabS1.docx]

**Table S1.** Dataset A subsample of patients from DiLiddo et al, 2020^a^ was selected to provide a framework of microbiomes from diseased patients. Conversely, a control set of sequences from healthy adults was downloaded from the SRA database^b^.

| **Sample ID** | **Category** | **Gender** | **AGE** | **Status** | **Copatology** |
| --- | --- | --- | --- | --- | --- |
| CD-9M72 | CD | M | 72 | remission | Yes |
| CD-10M44 | CD | M | 44 | remission | Yes |
| CD-22M30 | CD | M | 30 | sick | No |
| CD-24F30 | CD | F | 30 | sick | No |
| CD-43M68 | CD | M | 68 | remission | Yes |
| CD-11F43 | CD | F | 43 | sick | No |
| UC-7M72 | UC | M | 72 | sick | Yes |
| UC-8M72 | UC | M | 72 | sick | Yes |
| UC-23M65 | UC | M | 65 | sick | No |
| UC-26M73 | UC | M | 73 | sick | No |
| UC-39M48 | UC | M | 48 | remission | Yes |
| UC-40M80 | UC | M | 80 | sick | Yes |
| UC-41M61 | UC | M | 61 | remission | Yes |
| UC-42F52 | UC | F | 52 | sick | Yes |
| SRR4457136 | Healthy | NA | NA | healthy | No |
| SRR4457153 | Healthy | NA | NA | healthy | No |
| SRR4457155 | Healthy | NA | NA | healthy | No |
| SRR4457156 | Healthy | NA | NA | healthy | No |
| SRR4457163 | Healthy | NA | NA | healthy | No |
| SRR4457164 | Healthy | NA | NA | healthy | No |
| SRR4457165 | Healthy | NA | NA | healthy | No |
| SRR4457166 | Healthy | NA | NA | healthy | No |
| SRR4457167 | Healthy | NA | NA | healthy | No |
| SRR4457187 | Healthy | NA | NA | healthy | No |
| SRR4457188 | Healthy | NA | NA | healthy | No |
| SRR4457189 | Healthy | NA | NA | healthy | No |

1. Di Liddo R, Piccione M, Schrenk S, Dal Magro C, Cosma C, Padoan A, et al. S100B as a new fecal biomarker of inflammatory bowel diseases. Eur Rev Med Pharmacol Sci. 2020 Jan;24(1):323–32
2. Kodama Y, Shumway M, Leinonen R, International Nucleotide Sequence Database Collaboration. The Sequence Read Archive: explosive growth of sequencing data. Nucleic Acids Res. 2012 Jan;40(Database issue):D54-56
