# Supplementary material for: In Silico Evaluation of Putative S100B Interacting Proteins in Healthy and IBD Gut Microbiota
Source: Cells. 2020 Jul 15;9(7):1697. doi: 10.3390/cells9071697 (PMC7407188; doi:10.3390/cells9071697)
Supplement: Supplementary file 1 [file cells-09-01697-s001.zip › cells-805132_Supplementary material/SuppMat-30giugno2020_TabS2.docx]

**Table S2**. **Reads Count**. Sequences input data and reads polishing.

| sampleid | Sequence count | input | filtered | denoised | merged | non-chimeric |
| --- | --- | --- | --- | --- | --- | --- |
| CD-9M72 | 8082 | 63783 | 43100 | 43100 | 14069 | 3203 |
| CD-10M44 | 8472 | 59586 | 32424 | 32424 | 6206 | 1872 |
| CD-22M30 | 11283 | 63872 | 32838 | 32838 | 1747 | 1186 |
| CD-24F30 | 11023 | 68571 | 50920 | 50920 | 19878 | 4204 |
| CD-43M68 | 10308 | 71911 | 49214 | 49214 | 12280 | 3307 |
| CD-11F43 | 1900 | 21705 | 5419 | 5419 | 3020 | 1611 |
| UC-7M72 | 7478 | 74035 | 48851 | 48851 | 13840 | 3214 |
| UC-8M72 | 11261 | 81347 | 57843 | 57843 | 20492 | 3644 |
| UC-23M65 | 11241 | 53891 | 30005 | 30005 | 2483 | 2239 |
| UC-26M73 | 8698 | 37114 | 23060 | 23060 | 2203 | 1568 |
| UC-39M48 | 12196 | 57333 | 36585 | 36585 | 3728 | 2336 |
| UC-40M80 | 10737 | 57541 | 39374 | 39374 | 13315 | 3847 |
| UC-41M61 | 12220 | 67706 | 39397 | 39397 | 7243 | 4211 |
| UC-42F52 | 8276 | 33833 | 18556 | 18556 | 3356 | 2260 |
| SRR4457136 | 5074 | 120923 | 97825 | 97825 | 85771 | 4726 |
| SRR4457153 | 2236 | 28123 | 21147 | 21147 | 10297 | 1644 |
| SRR4457155 | 1109 | 16282 | 12672 | 12672 | 8957 | 1146 |
| SRR4457156 | 1352 | 45243 | 37315 | 37315 | 23339 | 1264 |
| SRR4457163 | 2287 | 62654 | 45906 | 45906 | 35245 | 2299 |
| SRR4457164 | 1574 | 22398 | 16973 | 16973 | 11495 | 1130 |
| SRR4457165 | 2726 | 28910 | 22535 | 22535 | 5773 | 1347 |
| SRR4457166 | 4955 | 95258 | 78586 | 78586 | 40325 | 3602 |
| SRR4457167 | 1184 | 23805 | 17875 | 17875 | 11056 | 1303 |
| SRR4457187 | 4116 | 45759 | 37792 | 37792 | 26915 | 3385 |
| SRR4457188 | 2642 | 36398 | 29923 | 29923 | 23387 | 2524 |
| SRR4457189 | 2210 | 36347 | 29766 | 29766 | 23015 | 1992 |
