# Supplementary material for: In Silico Evaluation of Putative S100B Interacting Proteins in Healthy and IBD Gut Microbiota
Source: Cells. 2020 Jul 15;9(7):1697. doi: 10.3390/cells9071697 (PMC7407188; doi:10.3390/cells9071697)
Supplement: Supplementary file 1 [file cells-09-01697-s001.zip › cells-805132_Supplementary material/SuppMat-30giugno2020_TabS3.docx]

**Table S3**. Microbiome Composition at Phylum Level. A: Unassigned; B: Euryarcheota (Archea); C: Kingdom Bacteria; D Actinobacteria; E: Bacteroidetes; F: Firmicutes, G: Fusobacteria; H: Gemmatimonadetes; I: OD1; J: Planctomycetes; K: Proteobacteria; L: Synergistetes; M: Verrucomicrobia; N: Thermi.

| Sample | A | B | C | D | E | F | G | H | I | J | K | L | M | N |
| --- | --- | --- | --- | --- | --- | --- | --- | --- | --- | --- | --- | --- | --- | --- |
| CD-9M72 | 0.3 | 0.0 | 3.6 | 3.0 | 34.1 | 24.8 | 0.0 | 0.0 | 0.0 | 0.0 | 31.4 | 0.0 | 2.9 | 0.0 |
| CD-10M44 | 1.0 | 0.0 | 6.3 | 0.3 | 49.2 | 15.3 | 0.3 | 0.0 | 0.0 | 0.0 | 26.0 | 0.0 | 1.6 | 0.0 |
| CD-22M30 | 1.1 | 0.0 | 2.9 | 0.2 | 2.4 | 4.2 | 0.0 | 0.0 | 0.0 | 0.0 | 89.2 | 0.0 | 0.0 | 0.0 |
| CD-24F30 | 0.3 | 0.0 | 2.6 | 3.0 | 54.7 | 32.2 | 0.0 | 0.0 | 0.0 | 0.0 | 7.2 | 0.0 | 0.0 | 0.0 |
| CD-43M68 | 0.2 | 0.0 | 1.2 | 4.3 | 17.3 | 25.0 | 0.0 | 0.0 | 0.0 | 0.0 | 51.9 | 0.0 | 0.0 | 0.0 |
| CD-11F43 | 0.0 | 0.0 | 0.0 | 0.0 | 0.0 | 49.0 | 0.0 | 0.0 | 0.0 | 0.0 | 51.0 | 0.0 | 0.0 | 0.0 |
| UC-7M72 | 0.3 | 0.0 | 10.3 | 3.9 | 30.6 | 21.1 | 0.0 | 0.0 | 0.0 | 0.0 | 31.0 | 0.0 | 2.9 | 0.0 |
| UC-8M72 | 0.7 | 0.0 | 2.2 | 6.5 | 49.6 | 21.7 | 0.0 | 0.0 | 0.0 | 0.0 | 18.8 | 0.0 | 0.5 | 0.0 |
| UC-23M65 | 1.5 | 0.0 | 8.7 | 1.1 | 5.8 | 7.0 | 0.0 | 0.0 | 0.0 | 0.0 | 75.4 | 0.0 | 0.4 | 0.0 |
| UC-26M73 | 0.5 | 0.0 | 4.2 | 0.6 | 7.4 | 11.8 | 0.0 | 0.0 | 0.0 | 0.0 | 75.5 | 0.0 | 0.0 | 0.0 |
| UC-39M48 | 1.1 | 0.0 | 6.4 | 1.2 | 8.8 | 6.5 | 0.0 | 0.0 | 0.0 | 0.0 | 74.3 | 0.0 | 1.6 | 0.0 |
| UC-40M80 | 0.7 | 0.0 | 4.2 | 2.3 | 16.3 | 33.4 | 0.0 | 0.0 | 0.0 | 0.0 | 42.4 | 0.0 | 0.8 | 0.0 |
| UC-41M61 | 2.2 | 0.0 | 14.4 | 1.3 | 49.1 | 12.9 | 0.0 | 0.0 | 0.0 | 0.0 | 20.2 | 0.0 | 0.0 | 0.0 |
| UC-42F52 | 0.4 | 0.0 | 8.8 | 1.9 | 8.0 | 13.6 | 0.0 | 0.0 | 0.0 | 0.0 | 67.1 | 0.3 | 0.0 | 0.0 |
| SRR4457136 | 0.0 | 0.0 | 2.2 | 45.4 | 0.0 | 49.6 | 0.0 | 0.0 | 0.0 | 0.0 | 2.8 | 0.0 | 0.0 | 0.0 |
| SRR4457153 | 0.0 | 0.0 | 0.5 | 57.3 | 0.4 | 15.7 | 0.0 | 0.0 | 0.0 | 0.0 | 26.1 | 0.0 | 0.0 | 0.0 |
| SRR4457155 | 0.0 | 0.0 | 0.0 | 24.5 | 0.0 | 75.5 | 0.0 | 0.0 | 0.0 | 0.0 | 0.0 | 0.0 | 0.0 | 0.0 |
| SRR4457156 | 0.0 | 0.0 | 0.9 | 13.3 | 0.0 | 44.7 | 0.0 | 0.0 | 0.0 | 0.0 | 41.1 | 0.0 | 0.0 | 0.0 |
| SRR4457163 | 0.0 | 0.3 | 0.0 | 23.2 | 0.0 | 75.3 | 0.0 | 0.0 | 0.0 | 0.0 | 0.0 | 0.0 | 1.2 | 0.0 |
| SRR4457164 | 0.0 | 0.0 | 1.7 | 52.9 | 16.8 | 9.0 | 0.0 | 3.2 | 0.0 | 2.7 | 11.0 | 0.0 | 1.5 | 1.3 |
| SRR4457165 | 0.0 | 0.0 | 0.4 | 33.1 | 0.0 | 61.3 | 0.0 | 0.0 | 0.0 | 0.0 | 5.2 | 0.0 | 0.0 | 0.0 |
| SRR4457166 | 0.0 | 0.0 | 0.6 | 15.8 | 0.0 | 40.6 | 0.0 | 0.0 | 0.0 | 0.0 | 41.9 | 0.0 | 1.2 | 0.0 |
| SRR4457167 | 0.0 | 0.0 | 0.0 | 63.6 | 0.0 | 10.5 | 0.0 | 0.0 | 0.0 | 0.0 | 25.9 | 0.0 | 0.0 | 0.0 |
| SRR4457187 | 0.0 | 0.0 | 4.3 | 6.6 | 2.9 | 82.0 | 0.0 | 0.0 | 0.0 | 0.0 | 3.6 | 0.0 | 0.7 | 0.0 |
| SRR4457188 | 0.0 | 0.0 | 0.4 | 0.0 | 1.3 | 88.0 | 0.0 | 0.0 | 0.0 | 0.0 | 10.3 | 0.0 | 0.0 | 0.0 |
| SRR4457189 | 0.0 | 0.0 | 0.6 | 24.9 | 0.0 | 72.4 | 0.0 | 0.0 | 0.0 | 0.0 | 2.1 | 0.0 | 0.0 | 0.0 |
