# Supplementary material for: In Silico Evaluation of Putative S100B Interacting Proteins in Healthy and IBD Gut Microbiota
Source: Cells. 2020 Jul 15;9(7):1697. doi: 10.3390/cells9071697 (PMC7407188; doi:10.3390/cells9071697)
Supplement: Supplementary file 1 [file cells-09-01697-s001.zip › cells-805132_Supplementary material/SuppMat-30giugno2020_TabS4.docx]

**Table S4**. Microbiome Composition at Genus Level. Genus reported in table have been used to query the Uniprot database

| Genus | CD | UC | CT |
| --- | --- | --- | --- |
| Akkermansia | 0.83 | 0.88 | 0.47 |
| Bacteroides | 30.91 | 23.01 | 0.69 |
| Bifidobacterium | 0.34 | 0.91 | 30.30 |
| Blautia | 0.24 | 0.13 | 0.01 |
| Butyricicoccus | 0.04 | 0.32 | 0.02 |
| Clostridium | 10.62 | 5.47 | 30.48 |
| Collinsella | 1.06 | 1.62 | 0.32 |
| Dorea | 0.47 | 0.69 | 0.42 |
| Faecalibacterium | 0.25 | 1.52 | 9.18 |
| Lactobacillus | 0.07 | 0.34 | 0.44 |
| Pseudomonas | 38.51 | 49.28 | 0.16 |
| Roseburia | 1.64 | 2.39 | 1.24 |
| Ruminococcus | 0.69 | 0.93 | 0.19 |
| Shigella | 1.80 | 1.41 | 16.32 |
| Streptococcus | 0.53 | 0.21 | 7.36 |
| planctomycete | 0.01 |  | 0.14 |
| Coprobacillus | 0.09 |  | 0.01 |
| Enterococcus |  | 0.03 | 0.20 |
| Eubacterium |  | 0.42 | 0.35 |
| Bordetella |  | 0.01 |  |
| Carnobacterium |  | 0.05 |  |
| Cloacibacillus |  | 0.03 |  |
| Cupriavidus |  | 0.04 |  |
| Ilumatobacter |  | 0.01 |  |
| Mucilaginibacter |  | 0.04 |  |
| Slackia |  | 0.02 |  |
| vadinHB04 |  | 0.04 |  |
| Acidaminococcus | 0.38 | 0.26 |  |
| Alistipes | 0.60 | 0.13 |  |
| Actinobacillus | 0.07 | 0.13 |  |
| Allobaculum | 0.02 | 0.01 |  |
| Barnesiella | 0.20 | 0.35 |  |
| Bilophila | 0.04 | 0.01 |  |
| Brevundimonas | 0.11 | 0.19 |  |
| Eggerthella | 0.78 | 0.15 |  |
| Elizabethkingia | 0.02 | 0.05 |  |
| Halomonas | 0.11 | 0.19 |  |
| Massilia | 3.55 | 3.63 |  |
| Morganella | 0.49 | 0.03 |  |
| Odoribacter | 0.19 | 0.45 |  |
| Oscillospira | 0.38 | 0.58 |  |
| Parabacteroides | 0.52 | 0.53 |  |
| Paraprevotella | 0.14 | 0.66 |  |
| Pectinatus | 0.08 | 0.14 |  |
| Prevotella | 0.83 | 0.38 |  |
| Pseudoramibacter_Eubacterium | 0.10 | 0.08 |  |
| Shewanella | 0.48 | 0.40 |  |
| Sphingobacterium | 0.21 | 0.25 |  |
| Sphingomonas | 0.01 | 0.02 |  |
| Stenotrophomonas | 0.70 | 1.13 |  |
| Veillonella | 0.85 | 0.04 |  |
| Vibrio | 0.29 | 0.39 |  |
| Actinomyces | 0.02 |  |  |
| Corynebacterium | 0.01 |  |  |
| Lachnobacterium | 0.33 |  |  |
| Paraeggerthella | 0.17 |  |  |
| Pedobacter | 0.01 |  |  |
| Psychrobacter | 0.03 |  |  |
| Rhodococcus | 0.02 |  |  |
| Rothia | 0.03 |  |  |
| Xylella | 0.15 |  |  |
| Azomonas |  |  | 0.07 |
| Christensenella |  |  | 0.02 |
| Enterobacter |  |  | 0.10 |
| Erythromicrobium |  |  | 0.16 |
| Escherichia |  |  | 0.09 |
| Glycomyces |  |  | 0.08 |
| Haloferula |  |  | 0.03 |
| Hyphomonas |  |  | 0.04 |
| Methanobrevibacter | |  | 0.03 |
| Parapedobacter |  |  | 0.41 |
| Staphylococcus |  |  | 0.04 |
| Streptomyces |  |  | 0.51 |
| Turicibacter |  |  | 0.13 |
